# Peer Communication in Online Mental Health Forums for Young People: Directional and Nondirectional Support

**DOI:** 10.2196/mental.6921

**Published:** 2017-08-02

**Authors:** Julie Prescott, Terry Hanley, Katalin Ujhelyi

**Affiliations:** ^1^ School of Education and Psychology University of Bolton Bolton United Kingdom; ^2^ School of Environment, Education and Development University of Manchester Manchester United Kingdom

**Keywords:** adolescence, Internet, social media, mental health, qualitative research

## Abstract

**Background:**

The Internet has the potential to help young people by reducing the stigma associated with mental health and enabling young people to access services and professionals which they may not otherwise access. Online support can empower young people, help them develop new online friendships, share personal experiences, communicate with others who understand, provide information and emotional support, and most importantly help them feel less alone and normalize their experiences in the world.

**Objective:**

The aim of the research was to gain an understanding of how young people use an online forum for emotional and mental health issues. Specifically, the project examined what young people discuss and how they seek support on the forum (objective 1). Furthermore, it looked at how the young service users responded to posts to gain an understanding of how young people provided each other with peer-to-peer support (objective 2).

**Methods:**

Kooth is an online counseling service for young people aged 11-25 years and experiencing emotional and mental health problems. It is based in the United Kingdom and provides support that is anonymous, confidential, and free at the point of delivery. Kooth provided the researchers with all the online forum posts between a 2-year period, which resulted in a dataset of 622 initial posts and 3657 initial posts with responses. Thematic analysis was employed to elicit key themes from the dataset.

**Results:**

The findings support the literature that online forums provide young people with both informational and emotional support around a wide array of topics. The findings from this large dataset also reveal that this informational or emotional support can be viewed as directive or nondirective. The nondirective approach refers to when young people provide others with support by sharing their own experiences. These posts do not include explicit advice to act in a particular way, but the sharing process is hoped to be of use to the poster. The directive approach, in contrast, involves individuals making an explicit suggestion of what they believe the poster should do.

**Conclusions:**

This study adds to the research exploring what young people discuss within online forums and provides insights into how these communications take place. Furthermore, it highlights the challenge that organizations may encounter in mediating support that is multidimensional in nature (informational-emotional, directive-nondirective).

## Introduction

It has been suggested that 75% of all mental illness emerges before the age of 25 [1]. Mental health can impact all aspects of young people’s lives, including their relationships, educational attainment, and quality of life [2]. Despite the high prevalence of mental health in young people, this is not matched with the level of service use [3]. Indeed, getting young people to access health services can be a challenge, especially services related to mental health [4].

### Online Versus Offline Support

The Internet affords a number of features the offline world does not. Research has found many of these features are important for young people seeking health information and offering support [5]. One feature of the Internet is that social cues are limited [6], which allow users to engage with those with whom they would otherwise not do so in the offline world [7], and it produces a hyperpersonal interaction [8]. A reduction of social cues facilitated contact in an online mental health forum, thus overriding differences with peers, such as gender, ethnicity, and disability [9]. The study also found that anonymity of the mental health forums was important, as this increased self-disclosure of forum users [9]. The anonymous nature of the Internet encourages the disinhibition effect [10], in that, people disclose and reveal more about themselves online than they would offline. Disinhibition is viewed to have a positive effect in online mental health forums [11]. Indeed, the features of anonymity, accessibility, and control (in terms of self-disclosure: what and how much people disclose) enables the Internet to provide a safe place to seek help [12]. Online counseling support has been perceived to be more confidential by young people than offline [5,13], with fewer risks, such as parents being informed. The Internet not only provides access to content around issues but also access to people in a similar situation, with no geographical restrictions. It also allows people to offer support, both professional and/or peer support [14]. Therefore the Internet has the potential to help young people by reducing the stigma associated with mental health and enabling young people to access services and professionals which they may not otherwise have been able to access. In general, the Internet can offer young people support through professional and peer support, as well as provide a referral to appropriate services [15].

### Online Platforms: An Alternative to Face-to-Face Support

It is generally recognized that young people do not tend to access health services and health professionals, especially in regard to their mental health [4]. Reasons for this lack of access include the stigma associated with mental health issues [4], as well as issues around confidentiality and access [16]. The Internet plays a salient part in people’s lives, and it is not just utilized by certain populations or for certain applications. Although it is recognized that a digital divide still exists in terms of who has access and how people use the Internet [17], research suggests that in the United Kingdom there are few young people who do not use the Internet [17]. Furthermore, accessing health information online is viewed as a confidential and convenient way to access information, which young people may find difficult to access offline, and as such, the Internet has become a primary source for general information and health information to young people [16,18].

### What Does Previous Research Tell Us?

In regard to mental health, research has considered online forums for young people suffering a number of mental health symptoms and conditions; these include self-harm [19,20], depression [15], those with suicidal thoughts [21,22], and young people with mentally ill parents [23]. The way in which young people use online forums is of increasing interest to researchers. Help-seeking behaviors related to how individuals gain information and support around issues linked to their physical and mental health needs is of particular interest. A notable benefit of online support groups is that the groups are available 24/7, which enables users to receive support and advice when more traditional resources are unavailable [24]. Online support groups also offer the convenience of support from similar people from within their own homes [25]. Research has started to explore the effects of online support for young people with mental health problems and other health issues with results indicating that online forums provide them with emotional and informational support [15,26-28], they can decrease isolation and stress [15,28], and reduce symptoms such as depression [21] and self-harm [20]. In a study looking at the viability of online blogs as a research methodology for young people with arthritis, Prescott et al [29] found that the online environment gave them space and empowerment to express their own ideas and concerns. The young people in the study found blogging to be therapeutic and enabled them to provide detailed thoughts, feelings, and experiences beneficial to researchers of an often hard to reach sample. Indeed, earlier research of an online university mental health community found participants benefited from the therapeutic benefit of writing [11]. Ultimately, online forums have the potential to provide a private and emotionally safe environment; this is especially so for forums that are moderated [30]. Furthermore, online forums maybe particularly appealing to young males who tend to have lower levels of help-seeking behavior than their female counterparts [4].

### Peer Support

Research has found forums beneficial to young people as it provided a safe place for them to share, offer, and receive emotional and informational support, again supporting earlier research on the supportive nature of online forums [15]. Recent research investigating online forums for young people with cancer [26] found both the requests for support, and the support provided, were found to be either informational support (which included themes such as medical aspects of treatment, advice to relieve fear, side-effects, and diet) or emotional support (which included themes such as keep fighting, I know what it’s like, and non-cancer users providing support). Similarly, research found young people exchanged emotional and informational support, coping, and identity management issues online [27]. The study also found young people used the forum to discuss non-cancer related topics such as music, sports, and friendships. Through the discussion of non-cancer-related topics, the group was able to build a strong community and provide each other with a sense of normality. The study found that many young people with cancer felt they could not talk to their offline friends and many had lost friends since diagnosis. This suggests that young people may not have access to offline peer-to-peer support and proves a potential reason why young people may value online peer support. This is an important finding of online forums, since it is often thought or assumed that young people seek informal support from friends or family members more so than from formal support mechanisms [4]. If this informal support is not so easily available offline, then online forums have a potentially vital role in the lives of young people who are vulnerable, isolated, or in some way marginalized.

Online self-help groups can provide a sense of normality to young people [23]. Knowing others have been or are currently going through a similar situation provides a sense of being normal and provides the opportunity to share experiences and feelings [23]. Online forums can provide social contact and support as a supplement to, rather than a replacement of, traditional health services. It was also revealed that professional involvement in forums was a valued aspect [9]. Suggesting forums benefit from some form of professional moderation, a view supported by Webb et al [31] who found a moderated online forum for young people in Australia to be a positive, unique, and helpful experience for young people suffering mental health problems. However, despite the benefits of forums being moderated, in a study aimed at bringing young people and health professionals together via an online forum for young people who self-harm, health professionals did not actively engage in the forum [20]. Reasons for the nonengagement included lack of confidence, private-professional boundaries, role clarity, duty of care, and accountability. Nevertheless, despite this lack of engagement by health professionals, the young people in the research shared their experiences and developed a strong community online. More research needs to consider barriers health professionals may face reaching young people through online forums.

Help-seekers have been found to provide support for others, suggesting young people adopt dynamic roles when using online forums that allow them to tell their stories and develop an online community of support [22]. It has been recommended that online support groups and forums should encourage active involvement to facilitate emotional relief and distress [21]. Active participation in support groups involves posting initial messages, responding to others, or receiving replies [21].

McKiernan et al (2017) [32] found that a small percentage of forum users directly requested advice or information (directive queries) from others and concluded that forum users were either uncomfortable with or not interested in receiving suggestions on how to behave. Instead, they were more interested in other people’s experiences and opinions to help them make their own decisions regarding their actions (nondirective queries). Although it was uncommon for forum users to use directive queries, when considering both moderator and user queries, moderators were more likely to use nondirective queries to elicit experience and opinion suggesting a facilitative role. One major problem that young people could face when bonding online with other young people with mental health issues could be a negative mood induction (or triggering) due to hearing about difficulties in other people’s lives [33,34]. Other issues which may impact young people’s use of forums that needs to be taken into consideration include data security, technical issues, and user safety [35].

It is evident that online support groups can support and empower young people, help them develop new friendships, share personal experiences, communicate with others who understand, provide information and emotional support, and most importantly help them feel less alone and normal. Often online communities develop for young people who do not feel supported in the offline world or they may want to discuss issues they feel embarrassed to share with their offline friends due to risk of stigma or embarrassment [23,28,36,37]. It is, therefore, important to understand online mental health forums for young people with a variety of concerns and needs. Furthermore, it is essential to consider the way that individuals engage with these services and how individuals may be supported to receive and provide support.

The aim of this research is to gain an understanding of how young people use online forums for their emotional and mental health issues and to gain an insight into how they support each other through this forum that is part of a wider online counseling service. The data covers a 2-year period, providing rich in-depth analysis on the ways young people interact. Specifically, the project examined what the forum users discussed and how they sought support through this platform (objective 1). Furthermore, it looked at service users’ responses to initial posts to gain an understanding of how peer-to-peer support was provided (objective 2).

## Methods

Kooth is an online counseling service for young people aged 11-25 years experiencing emotional and mental health problems. It is based in the United Kingdom and provides support that is anonymous, confidential, and free at the point of delivery. Kooth offers young people a number of services including drop-in and one-to-one chats with fully trained counselors, a themed moderated message forum, a secure Web-based email, and an online magazine. Young people register on the site using an anonymous user name.

### The Forum Data

The themed moderated forums on Kooth are relationships, bullying, eating disorders, depression, self-harm, health, friends, family, and ideas for Kooth. Despite having themed forums, the dataset was initially analyzed afresh by the researchers, rather than keeping the data within the themes devised by the Kooth site. This was undertaken by the researchers so as to allow the data to lead the theming, an inductive approach to data analysis [38,39], and capture any areas not outlined in the organization’s framework. The quotes have been kept in full to give a transparent flavor of the communication.

Kooth provided the researchers with all the forum posts (1 dataset) between a 2-year period (initial posts, posted on the forum from December 12, 2013 to December 31, 2015) that resulted in a dataset of 622 initial posts, 3657 initial posts and responses, 8 moderator initial posts, and 113 moderator posts and responses. The dataset was then divided into 2 datasets for deeper analysis of posters as well as the responses. These will briefly be discussed in turn below.

The first dataset was the initial posts (the help-seekers’ posts; 622 posts), analyzed to understand the issues young people wanted to discuss or seek support for, as well as to understand how young people started conversations and posts on this site (objective 1).

The second dataset included both the initial posts from individuals we will refer to as posters and the responses to each of the posts from individuals we will refer to as responders. This was the largest dataset with 3657 posts analyzed, to gain an understanding of how young people provided each other with peer-to-peer support (objective 2). Young people set up a username to post on the forum. Since the young people are posting on the forum for genuine, rather than research purposes, the research team decided not to include any username details so that all quotes are unidentifiable and completely anonymous. Due to the anonymity of the site, no participant information is available.

### Data Analysis

In total, the overall datasets included 160 unique posters (dataset 1) and 1320 unique posts (posters—the person who initiated the post on the forum, and responders—people who responded or commented on the initial post) overall (dataset 2). The most comments on a post was 170, and the least was one (a few posts received no response); however, most posts received between two and ten comments. All data was analyzed using Nvivo version 10 software (QSR International). The qualitative approach used to analyze each of the datasets was thematic analysis [38]. All authors reviewed the themes and discussed at length to refine the themes, the subthemes, and the hierarchy. This refining process helps to demonstrate the overall trustworthiness within the data analysis [40]. To ascertain and increase intercoder reliability and the reliability of the results, the raw data was read by all authors to develop a coding framework and code book. Once the code book was established, author KU, employed as a research assistant on the project, coded the datasets accordingly.

### Ethical and Research Approvals

Approval for the study was received by University of Bolton Research Ethics Committee in January 2016. 

## Results

The results will be discussed in terms of addressing objective 1 from dataset 1 first, then objective 2 from dataset 2. The analysis revealed the following themes and topics (Table 1) related to the two objectives and datasets. Table 1 shows the main themes, topics, and an example quote from the interview analysis.

### What Young People Discuss

Young people discussed a range of mental health (such as anxiety, depression, panic attacks, eating disorders, suicide, and self-harm) and physical health issues (such as pregnancy, periods, cancer, diabetes, and epilepsy). They also discussed numerous issues related to interpersonal relationships, which included friendships, sexual relationships, family issues, death, isolation or loneliness, and bullying. Other issues that were frequently discussed included those related to school (anxious about school, too much school work, and not fitting in), sexuality (coming out and confused), and identity issues (self-esteem or confidence, appearance, and being different). Although this is not a complete list of the issues discussed, it is apparent that the themes suggested on the forum do not limit, or in any way restrict, the discussions on the forum.

It was also observed that the young people in the forum frequently posted and responded to noncounseling-related issues. For instance, there are a number of posts discussing topics such as music, film, and pets, and there are plenty of jokes on there too. These discussions suggest a sense of community and friendship, aside from the problem specific supportive element of the forums, being developed on the forum and also the sense of wanting to share. Interestingly, there is discussion of Internet and social media use, with security or privacy issues, the benefits of anonymity, as well as the ability and benefits of sharing with others online in a similar situation as highlighted in the example quote in Table 1.

### How Young People Seek Support

In terms of how the young people seek support, posters tended to approach this in one of two ways. Either by direct request for advice, with a themed heading followed by a post with more detail about the specific advice they require, or a direct question within the post itself. The example quote in Table 1 is by a young person asking for advice on how they can cope with panic attacks. Indeed, the title header of the post states this is what the poster is seeking advice on, which is then followed by more detail on the issue and further detail on the specific advice they are seeking. In this instance it’s about seeking advice on how to deal with them, in particular how to cope during class.

Other ways the young people sought advice was by finding other young people on the forum who shared similar feelings or were in a similar situation to themselves. Through doing this, young people were able to start a dialogue relevant to their concerns and issues and hopefully gain support through the responses received. Many posters provide detailed background information when seeking support or advice as shown in the example quote in Table 1.

**Table 1:** Objectives and themes.

Objectives				Example quote
Objective 1				
**What young people discuss**		Mental health issues, physical health issues, interpersonal relationships, school-related issues, sexuality, identity issues, noncounseling-related issues	I use Instagram to talk to people who have the same sort of feelings as me. I think it is a good thing because it has helped me get a better understanding of what exactly it is that I’m feeling and sort of a sense of security because I know I have people I can talk to and I know I’m not alone with this. I think problems occur when people pretend to be someone that they aren’t...	
**How young people seek support**		Direct request for advice	How to cope with panic attacks? -Lately all my panic attacks have been getting worse and extremely frequent. I’m getting them in school especially. How do you cope with them personally? And, what do you do if you get them in class? Their really getting in the way of everything, and I don’t know how to deal with this anxiety anymore.	
		Seeking others in a similar situation or with similar feelings	My parent has depression, advice? So I’m 17 and old enough to understand what and how bad depression can be. But I never expected for my mum to be diagnosed with it. You can only do so much; I’m 17 and still find it a daily struggle to watch someone I love and care for with every breath go through such an awful illness. What are your thoughts do you have any advice?	
		Offering support	Adoption: Hi, I am X 2754. I am adopted and don’t always believe my parents love me. Because of this, I know it’s hard being adopted and people who are adopted all understand this. We may all experience separation and flashback anxiety; I have terrible anxiety, always constantly thinking my birth parents are trying to track me down and are watching my every move. It’s hard to overcome these anxieties. I just think to myself, “try to get on with life; you only live once, don’t let it overtake you when you’re still young also don’t waste your life because you’re in this situation, make the most of life whilst you still can. Never think that your family now don’t love you; they OBVIOUSLY DO, OTHERWISE THEY WOULDN’T HAVE ADOPTED YOU IN THE FIRST PLACE.” I hope I help people with anxiety and panic attacks who suffer from these because of being adopted that you only live once, and don’t let it be misery but happiness. Good luck to you all; let adoption strive forward.	
Objective 2				
**Emotional support**		Nondirective emotional support	I can completely relate to all of these comments. I have attempted before but now I feel like they don’t treat my feelings seriously like if I’m not hurting or trying to take my life although it still comes to mind that I’m not feeling a certain way...nobody around me understands me. But on kooth, I feel like the community does. At least on here, there are people who can say that it isn’t just you Yano. You don’t have to go it alone. We take you seriously. You are exactly who you are supposed to be and you fit perfectly into your own spot in life, even though it may not feel like it, you are important to a lot of people.	
		Directive emotional support	I have had a fair few panic attacks in my life and I understand how HORRIBLE they are. My first panic attack was when my insomnia was getting seriously bad and I started panicking about not sleeping and I just lost it. From my personal experience I would recommend, 1, talking to someone about it, what you’re going through, helpful ways of dealing with it, and also to get your worries off your chest, which can help a lot trust me. 2, tell people. The more people know, the more people can help you when you are in that state. But only tell people you know very well and trust.	
Informational Support		Nondirective informational support	I found papyrus and the Samaritans particularly good as I emailed them when I was having a hard time living and still am but they’re very helpful and it’s not just for suicide you can talk about anything with them and they’ll listen and they’re good to vent for. As I’ve been self-harming for three years and only recently noticed how bad I’ve gotten and how different I am to others and they helped me get to the root of my problems and gave me the courage to see a doctor as did the counsellors on kooth who then gave me a cahms referral [camhs stands for Child and Adolescent Mental Health Services, a specialist NHS service].	
		Directive informational support	Can you confront your friends about this? Or find an interest you both share? You don’t need to be a normal teenager, and if they’re treating you like this, then they are not the friends they seem. It may seem easier said than done but how about joining a club to make new friends. A typical response, yes, but joining a club is one of the best ways to meet people who share your interests. And if there are no clubs in your area that support your interests, how about starting one. As for your diabetes, if you can’t confront your father about this, is there someone who can? Can you contact your mother in any way? Or perhaps you could visit your local GP surgery or school nurse for some advice on coping with your illness. Good luck.	

Not all of the posters sought support; many posters instead offered support in their opening post. Through starting a communication on the forum on a specific topic relevant to them allows others to comment and receive support. Responses to advice giving posts include seeking further advice and further developing some kind of friendship. For example, it can be seen from the following response that the opening, initial post provided an opportunity for others to seek advice and support:

I have a friend that is adopted and sometimes when there upset I don’t know what to say something and I am scared to say something about my home life in front of her I don’t know how to cheer her up when she is upset Can you help Btw I think you’re really brave telling this.

Many young people provided support from personal experience, telling their story in a cautionary way for others to learn from. The following poster is sharing their experience of self-harm and informing others what it is like to self-harm; discussing the emotional battle they have gone through, the physical implications, and warning people against it, as in the quote below:

I don’t know how to deal with it-Hi I feel so horrible about self-harm. At the minute I’m really trying to stop cutting myself so much because it suddenly hit me hard when I looked at myself in the mirror. My legs are full of scars a deep purple scar on my arm and there is now a new one on my wrist. Okay the one on my arm just looks like an accident, but I now know how it would look to other people. No matter how hard I try, I can’t cover or fade them. The depression and anxiety around it has grown worse, and I think more about cutting. Please guys, if you have not yet self-harmed and you are considering it, just think could you look at your injury every day? Do you want to create a cycle? If you want to get better, just start by asking yourself these questions because doing something like self-harm has harsh consequences that might make things worse.

In light of previous research, it could be posited that posters offering support rather than seeking it are perhaps gaining from the therapeutic benefits of writing and sharing their experiences with others in this online context. The encouraging responses in terms of how their advice has been positively received may also provide posters with emotional support as well as through the gratitude observed in responses, such as, *Your advice is very kind thank you for posting this,* and *Thank you for your last piece its important people know that.* However, the data does not allow us to determine if this is an actual goal of the posters.

### Support on the Forum

**Figure 1:**
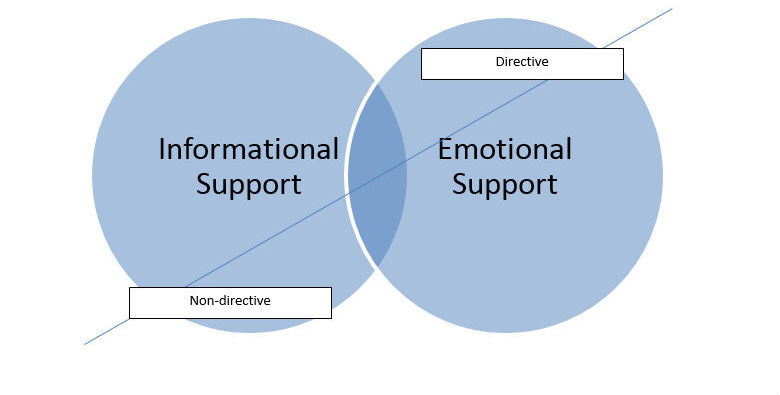
Type of Support.

The main forms of support provided by responders on the forum can be classified as emotional and informational support. When examining this further, this emotional and informational support can be viewed as being provided either in a directive or nondirective way. For instance, nondirective emotional support would involve the responder sharing an experience and not explicitly following this with advice, whereas directive emotional support would involve the responder sharing an experience while also advising on what to do. Similarly, nondirective informational support involves the responder sharing information and not providing explicit advice, while when offering directive informational support the responder shares information while also suggesting the poster do something. Figure 1 illustrates the support provided from the forum data analysis.

#### Emotional Support

The emotional support theme includes responses such as you are not alone, things will get better, it’s not your fault, don’t worry about what people say or think, empathizing, sympathizing, understanding, how to grow from the experience, it’s normal, you’re perfect the way you are, and I’m here if you want to talk. There were also a number of encouraging phrases used such as be yourself, do what is best for you or makes you happy, believe in yourself, be brave, have confidence in yourself, and look to the future. Some of these responder posts are encouraging but cautious. For instance, the following responder, despite being emotionally supportive and providing encouragement to the poster, is suggesting they wait until they are older before they make any decisions, as in the quote below:

Do what comes naturally to you I’d say; wait a few years after school and puberty and your hormones. I know I probably sound like I’m just saying it’s a phase but honestly it could well be.

#### Nondirective Emotional Support

A lot of the responders shared their own personal experiences in a nondirective way to provide or offer emotional support to posters. In the example quote in Table 1, the initial poster had described feeling alone and describes suffering from depression with little support from offline sources. One of the responders provides nondirective emotional support, expressing how they too have previously felt in a similar situation and how the community online can help as it had helped them previously. The response is supportive and encouraging. It is encouraging in that the responder is supporting the poster to reach out to people online, expressing that they will be taken seriously in this online environment, and they are a community who understands and supports. There are many examples of this type of emotional support where the support is provided by the responders showing empathy and knowledge through their own experience of being in a similar situation or experiencing similar experiences or emotions.

#### Directive Emotional Support

The forum data contained less directive emotional support compared with nondirective; however, this type of emotional support was provided on occasion. The blurred boundaries between nondirective and directive support were evident in the data. The example quote in Table 1 provides an example of a responder commenting on a post with nondirective emotional support. The responder in the first instance is describing how they understand due to having themselves gone through a similar situation or experience, in this case, panic attacks. The responder then moves on to provide the poster with some more directive practical advice by suggesting they talk to someone about it, highlighting how talking about it had helped them in the past.

#### Informational Support

Informational support in the form of practical advice is provided frequently on the site. For example, how to deal with a condition or issue, to provide others with information on a topic, or where to seek further help or answers.

#### Nondirective Informational Support

Responders frequently provided information to posters that they themselves found useful when they were going through similar difficulties based on experiences and emotion. Responders tended to provide a level of personal detail to provide the informational support or suggestions on how to help the situation as highlighted in the quote in Table 1. It can be seen from this quote that the responder is not just suggesting to the poster to contact the organizations mentioned but is instead telling the poster how useful they found them when they were going through a difficult time. The responder is disclosing to the poster their personal story to provide this information rather than simply stating who may be able to help.

#### Directive Informational Support

Although there are frequent examples of nondirective informational support as exemplified above, it was more frequently observed that young people offered more directive forms of informational support. In the example provided in Table 1, the poster had discussed having a number of issues and not knowing where or who to turn to. One responder offers support in the form of directive informational support suggesting the people they may be able to contact such as friends, general practitioner (GP) surgery, or school nurse. In contrast to the nondirective informational support, this response has no personal story to support why they are suggesting the advice they are or indeed why they think the poster should contact certain people as evidenced from the example quote for the nondirective informational support. It is however clear that the advice, although lacking in any personal background story, or experience, is empathic in nature, and the responder is trying to provide the poster with some encouragement and support.

Despite the variety of issues discussed on the forum, analysis revealed that some mental health or emotional issues tended to get a particular response. Table 2 illustrates the issues that received specific styles of response or support.

**Table 2:** Issues and support received.

Issues	Support theme	Approach
Transgender	Emotional support, for example, be yourself	Nondirective
Anxiety and panic attacks	Informational support	Directive
Self-harm	Emotional and informational	Directive
Bullying	Emotional support: not alone	Nondirective
Pregnancy related	Informational support	Directive

## Discussion

### Principal Findings

The study examined what the forum users discussed and how they sought support and found that forum users were provided with both informational and emotional support. This is consistent with previous research findings [15,26-28]. However, the findings from this large dataset reveal that this informational or emotional support can also be considered as coming from one of two approaches; directive or nondirective. The nondirective approach refers to young people providing others with either informational or emotional support by sharing their own experiences. The majority of responses were from young people in a similar situation, sharing practical advice or information on what works or worked for them, or what they have been recommended by health professionals. In light of previous research findings [25], through sharing similar experiences, the young forum users gained an understanding that they may not have received from their offline friends and family. The sharing of experiences also appears to provide empathy, the feeling of being less isolated, and that individuals are not alone in their situation; helping to provide some sense of normality and thus providing emotional support. The directive approach, in contrast, is associated with offering more practical advice. In using this approach, responders were able to help others with whom they may not share experiences but they still want to, and feel able to, offer support; adding a new dimension to peer support online.

The nondirective responses observed in the forum interactions might be considered in a similar vein to the therapeutic style advocated by person-centered therapists [41]. From this perspective, therapists are encouraged to be mindful of the client’s frame of reference, and thus, be more tentative in making assumptions about a person’s particular circumstances. Stereotypically, and we acknowledge the complexity inherent in such dynamics, responses from therapists might reflect back and summaries the words of those being helped, rather than explicitly direct the client to new ideas or ways of thinking. In all instances, therapeutic interventions would not be offered with the intention to steer the conversation to new territories but to communicate understanding and empathy. Here we note that the responses from the users in the forums varied greatly in style, but many might be construed in this nondirective way. Thus the respectful, caring tone of the responses, rather than the practical directive advice, can therefore be viewed as an important resource in such environments.

Considering the directive or nondirective form of support provides a novel approach to understanding how young people seek (posters) and provide (responders) support via an online forum based on their experiences. Posters tended to use more directive approaches when seeking support. However, even when directly requesting advice, posters often explained their personal situation to request advice in the first instance. What is most striking from the analysis is that many posters were not seeking support but instead were posting on the forum to offer what appears to be support to others on the forum. This could be linked to the disinhibition effect of the Internet [10] and the positive effects of disinhibition of mental health forums [11]. Offering support that has not been requested could be an avenue for young people to share their experiences on the site. This may provide those posters with the therapeutic benefits of writing about their difficulties [11,29], very much suggesting a dynamic approach to online support [22]. They may also share in the aim of potentially supporting others in similar positions. It would be interesting to understand this approach by young people in more depth, this perhaps being a feature of young people’s use of online support as a less conventional approach.

The implications of the directional and nondirectional approach of forum usage could highlight to young people how their views and personal experiences may be used and helpful to other young people. Moderators of such online forums might want to consider the impact of postings that are more directive in nature, since they may not always be accurate and be based more on opinion than evidence or fact. Furthermore, if someone is asking for advice and the response they receive is reflection, then moderators may want to mediate so that the posters receive what they have requested. In terms of future research, it would be interesting to explore what people gain from the different response styles. It is interesting that certain issues primarily received a certain type of response, whether that be emotional or informational support and whether that comes from a directive or nondirective approach. Reasons for this would help researchers and health professionals understand forum use and health information seeking of young people and enable the development of future online services.

Findings revealed the young people using the forum were not limited to topic specific issues. It was also observed that different issues tended to receive different types of support and responses. This is particularly interesting and somewhat important, since much of the literature investigating the use of online forums focuses on issue or topic specific forums, such as self-harm [20,19] or depression [15]. Young people could discuss anything that concerned them, even discussing things that were not directly a health or mental health concern, such as music, films, and pets —a factor that perhaps reflects the holistic or humanistic position taken by Kooth. In these instances, music was openly discussed as being therapeutic, with the sharing of new music or favorite music with others helping to develop communities and provide what they considered to be uplifting music to others. In a similar vein, other topics were discussed and seemed to provide young people with a platform to talk to each other and build a community in a safe and friendly (moderated) environment. Jokes were often told, again presumably to build a fun community. This noncounseling discussion on the site supports previous research [26]; these discussions suggest a sense of community and friendship, aside from the problem specific supportive element of the forums, being developed on the forum and also the sense of wanting to share.

Interestingly, there is discussion of Internet and social media use, with security or privacy issues, the benefits of anonymity, as well as the ability and benefits of sharing with others online in a similar situation. Young people discussed the Internet more generally and social media such as Instagram and Facebook and how they used them to connect with others and offer each other advice on online safety. Supporting previous findings related to the affordances of the Internet benefiting young people seeking support online [5,13-15,31]. This may also potentially link to previous literature [27], suggesting that young people feel comfortable discussing issues outside of the topic areas not necessarily related to mental health issues.

### Strengths and Limitations

Despite researchers growing interest in the area, much of the research has been conducted on an adult population. Research that has considered young people has tended to be conducted with university-based counseling sites [4,11,15] or sites set up specifically for certain conditions such as self-harm or depression [20-22], whereas this study is based on a large dataset of forum data from an established and active online counseling service aimed specifically at young people, allowing young people to discuss any issues relevant to them. A major strength of the study is that the study was conducted using a large dataset of posts and responses. These were posted over a 2-year period to an active and well-established online forum providing support for young people with emotional and mental health issues. This large set of forum data enabled the researchers to gain a deeper understanding of how young people used the forum; what they discussed, how they sought help, and how they provided peer-to-peer support on the forum. In particular, a major finding of the study is the novel approach of the support young people provided; either directional or nondirectional. This finding should be recognized as an important attribute of peer-to-peer support provided by young people using the forum.

There were many different emotional and mental health issues discussed. It is evident that despite the site having themed topics of discussion, what is discussed on the forum is not restricted or limited to these themes, and it is apparent that young people feel comfortable discussing issues outside of these areas on this forum. This suggests that the forum provides a safe environment for young people to disclose, supporting previous findings in the area [12]. Perhaps due to a reduction in social cues and the anonymous nature of online communication enables this safe environment [9].

Despite the strengths, a number of potential limitations and challenges should be acknowledged. First, the forum data did not allow for the researchers to take into account any demographic considerations such as age, gender, or location of the users of the forum. Demographic considerations would provide further insight into who uses the forum for support. This information may establish any demographic groups who do not use the site. This information could prove useful for service providers in accessing and potentially reaching a wider demographic or target hard to reach young people who may not be aware of the site. However, due to the anonymity of the site, it was not possible for this study to gain such information.

It could also be beneficial for researchers and service providers to know how young people use forums such as Kooth in conjunction with other services the site offers, such as one-to-one counseling, as well as how they use the site in conjunction with other counseling and health services, both online and offline. In essence, a deeper understanding is required into why the young people use this particular site and if they use other services for support.

It was notable that the responses and style of support varied greatly, with some posts reflecting content that might not always be construed as positive, or in some cases accurate. Such a dynamic proves a challenge to service providers, with the assessment of when to moderate or censor posts proving a major challenge. Future research may consider the impact of the multidimensional nature (informational-emotional and directive-nondirective) of responses to posts and how posters receive them.

Another limitation of the study is that the forum data does not give any real indication of how the responses are received and how helpful the responses are to young people. Further work is needed to explore the advice received and how the support provided is utilized. It is evident from the dataset that posts are online for a significant period of time, with young people able to continue to read and respond to these posts years after the initial posting. It would therefore be interesting to understand how these archived posts continue to help and support young people.

A further limitation is the limited generalizability of the findings. The findings from the study may be applied to other contexts of a similar online setting for young people. Indeed the findings may resonate with how young people approach online mental health services and health information more generally online. It should also be noted that the findings are based on the online forum service offered by Kooth and that this is just one of the services Kooth provide for young people seeking support with mental health and emotional issues. Due to the anonymity of the site, the extent of the needs of the young people using the forum is unknown to the researchers. Furthermore, it is not possible to identify where else young people gain further support either via Kooth or through other services.

It has been noted that online forums are viewed more positively when moderated [9,31]. Further research may consider the role of the moderator and how they recognize, and indeed manage, any such issues that might arise.

More research on the views of the young people who frequently and actively use online forums may help consider the potential downside to online forums. Previous research [21] suggests that active participation increases the likelihood of gaining benefits from being involved in online forums. Further understanding of participation level in online forums would also add light to how people use and potentially benefit from them, especially from young people who may be considered “lurkers” rather than active users of such forums.

This research indicates that the variety of support approaches may have different benefits to different people with a wide range of emotional or mental health needs. Further research might therefore consider these differences in the adoption of these approaches.

### Conclusions

This study provides a unique insight into how young people seek and provide each other with online support for their emotional and mental health needs from a large dataset of online forum posts, adding to the developing body of research exploring the way young people interact in online forums. The research highlights the breadth of issues that young people discuss in online forums. Furthermore, it demonstrates how this medium can provide young people with a place to seek and provide emotional and informational support. In addition to this, it is interesting to observe that those using the site provide support in directive or nondirective ways, a factor that poses questions for those moderating such services. More research is clearly needed to examine the nuances of such interactions and community building processes.
